# 1-(5-Hydr­oxy-3-methyl-1-phenyl-1*H*-pyrazol-4-yl)ethanone: a new monoclinic polymorph

**DOI:** 10.1107/S1600536809001470

**Published:** 2009-01-17

**Authors:** Tasneem Ullah Sheikh, Misbahul Ain Khan, Muhammad Nadeem Arshad, Islam Ullah Khan, Helen Stoeckli-Evans

**Affiliations:** a Department of Chemistry, Islamia University, Bahawalpur, Pakistan; b Department of Chemistry, Government College of Education, Afzalpur, Azad Jammu & Kashmir, Pakistan; c Materials Chemistry Laboratory, Department of Chemistry, GC University, Lahore, Pakistan; d Institute of Physics, University of Neuchâtel, rue Emile-Argand 11, CH-2009 Neuchâtel, Switzerland

## Abstract

The title compound, C_12_H_12_N_2_O_2_, crystallized in the monolinic space group *P*2_1_/*n*, with two independent mol­ecules (*A* and *B*) in the asymmetric unit. This is in contrast to the first monoclinic polymorph reported [Cingolani *et al.* (2002 ▶). *Inorg. Chem.* **41**, 1151–116], which crystallized in the space group *C*2/*c* with one independent mol­ecule per asymmetric unit. The dihedral angles between the two rings differ slightly; in mol­ecule *A* it is 4.90 (11)° and in mol­ecule *B* it is 16.05 (13)°. In both mol­ecules, there is an intra­molecular O—H⋯O hydrogen bond involving the hydroxyl substituent and the carbonyl O atom of the adjacent acetyl group. In the crystal structure, mol­ecules *A* and *B* are linked *via* a C—H⋯N inter­action. There are also some weak C—H⋯π inter­actions involving the phenyl ring of mol­ecule *A* and H atoms of the acetyl groups of both mol­ecules.

## Related literature

For early literature on pyrazoles, see: Knorr (1883 ▶). For information on the pharamceutical properties of pyrazoles, see: Grimmett (1970 ▶). For the monoclinic *C*2/*c* polymorph of the title compound, see: Cingolani *et al.* (2002 ▶).
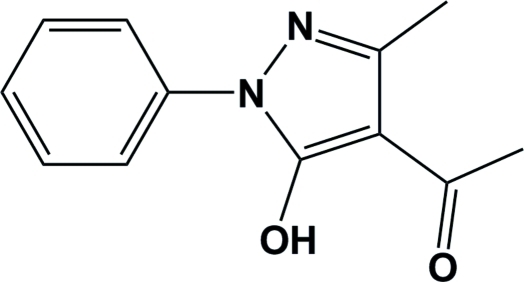


## Experimental

### 

#### Crystal data

C_12_H_12_N_2_O_2_
*M* *_r_* = 216.24
Monoclinic, 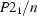

*a* = 13.8735 (7) Å
*b* = 9.2037 (4) Å
*c* = 18.3702 (8) Å
β = 110.100 (2)°
*V* = 2202.78 (18) Å^3^
*Z* = 8
Mo *K*α radiation
μ = 0.09 mm^−1^
*T* = 296 (2) K
0.34 × 0.22 × 0.16 mm

#### Data collection

Bruker Kappa APEXII CCD diffractometer
Absorption correction: none
24519 measured reflections
5500 independent reflections
2656 reflections with *I* > 2σ(*I*)
*R*_int_ = 0.047

#### Refinement

*R*[*F* ^2^ > 2σ(*F* ^2^)] = 0.051
*wR*(*F* ^2^) = 0.149
*S* = 0.97
5500 reflections
292 parameters
H-atom parameters constrained
Δρ_max_ = 0.18 e Å^−3^
Δρ_min_ = −0.14 e Å^−3^

### 

Data collection: *APEX2* (Bruker, 2002 ▶); cell refinement: *SAINT* (Bruker, 2002 ▶); data reduction: *SAINT*; program(s) used to solve structure: *SHELXS97* (Sheldrick, 2008 ▶); program(s) used to refine structure: *SHELXL97* (Sheldrick, 2008 ▶); molecular graphics: *PLATON* (Spek, 2003 ▶); software used to prepare material for publication: *SHELXL97*.

## Supplementary Material

Crystal structure: contains datablocks I, global. DOI: 10.1107/S1600536809001470/wn2305sup1.cif

Structure factors: contains datablocks I. DOI: 10.1107/S1600536809001470/wn2305Isup2.hkl

Additional supplementary materials:  crystallographic information; 3D view; checkCIF report

## Figures and Tables

**Table 1:** Hydrogen-bond geometry (Å, °)

*D*—H⋯*A*	*D*—H	H⋯*A*	*D*⋯*A*	*D*—H⋯*A*
O1—H1*O*⋯O2	0.82	1.85	2.546 (2)	142
O21—H21*O*⋯O22	0.82	1.83	2.531 (3)	142
C8—H8⋯N22^i^	0.93	2.56	3.489 (3)	177
C13—H13*C*⋯*Cg*2^ii^	0.96	2.66	3.533 (3)	150
C33—H33*B*⋯*Cg*2^ii^	0.96	2.98	3.774 (3)	141

Symmetry codes: (i) 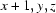; (ii) 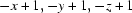. *Cg*2 is the centroid of the C6–C11 ring.

**Fig. 1.**
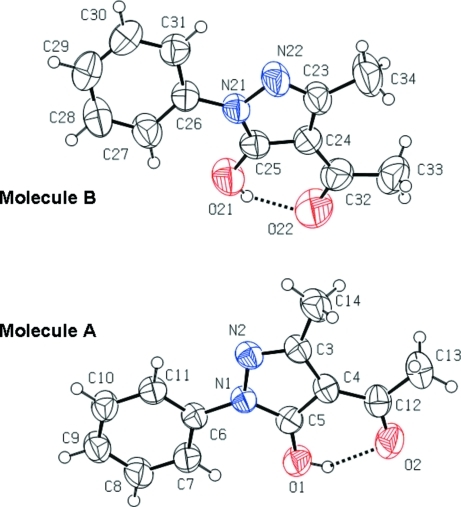
A view of the molecular structure of the two independent molecules (A and B), showing the displacement ellipsoids drawn at the 50% probability level and the intramolecular O—H···O hydrogen bonds as dashed lines. Hydrogen atoms are represented as spheres of arbitrary radius.

**Fig. 2.**
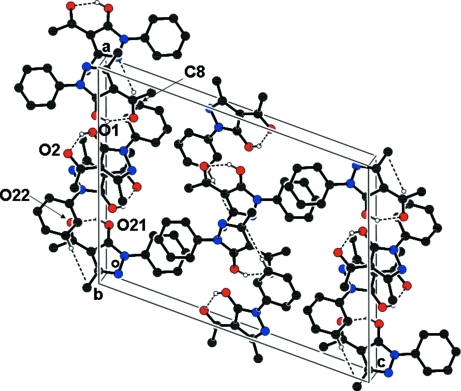
A view along the b axis of the crystal packing, showing the intramolecular O—H···H hydrogen bonds and the intermolecular C—H···N interactions as dashed lines. H atoms not involved in hydrogen bonding have been omitted for clarity.

## supplementary crystallographic information

### Comment

The history of pyrazoles began already in the late nineteenth century (Knorr, 1883). Pyrazole is isomeric with the biologically important imidazole ring system but, unlike imidazole, has fewer natural derivatives. The ring system is very stable and inert, and interest in such compounds stemmed from their applications as drugs, dyes and as anesthetics. They are also used as antioxidants in fuels but their major applications have been in the pharmaceutical (Grimmett, 1970) and agricultural industries. In view of the importance of pyrazole derivatives we have planned a systematic study of such compounds, and describe here the crystal structure of a new polymorph of the title compound.

It crystallized in the monoclinic space group P2_1_/n, with two independent molecules (A and B) per asymmetric unit (Fig. 1). This is in contrast to an earlier reported monoclinic polymorph, (Cingolani *et al.*, 2002), which crystallized in the space group *C*2/*c* with one independent molecule per asymmetric unit. The bond distances and angles in both polymorphs are very similar. The dihedral angles between the two rings differ slightly; in molecule A it is 4.90 (11)° and in molecule B it is 16.05 (13)°. The corresponding value in the other polymorph is 5.33 (10)°.

In both molecules (A and B), there is an intramolecular O—H···O hydrogen bond involving the hydroxyl substituent and the carbonyl O atom of the adjacent acetyl group (Table 1); this feature is also present in the *C*2/*c* polymorph. In the crystal structure, molecules A and B are linked *via* a C—H···N interaction (Fig. 2 and Table 1). There are also some weak C—H···π interactions involving the phenyl ring (centroid Cg2) of molecule A and some H atoms of the acetyl groups of both molecules (Table 1).

### Experimental

1-Phenyl-3-methyl-5-pyrazolone (7.5 g) was dissolved by heating in tetrahydrofuran (80 ml). Calcium hydroxide (12 g) was added and acetyl chloride (4 ml) was then added dropwise over a period of 1 min. The temperature increased during the first few minutes and the reaction mixture became a thick paste. This mixture was then refluxed for 30 min. The calcium complex of the title compound that had formed in the flask was decomposed by pouring the mixture into a dilute solution of HCl (100 ml). A dark brownish-red organic layer was obtained which was extracted using dichloromethane. The solvent was then removed by vacuum distillation and the solid obtained was washed with a little water and THF. Crystals of the title compound, suitable for X-ray analysis, were obtained by recrystallization from methanol/water (1:1, v:v).

### Refinement

The H atoms were included in calculated positions and treated as riding atoms: O—H = 0.83 Å, C—H = 0.93 - 0.96 Å with *U*_iso_(H) = k*U*_eq_(parent atom), where k = 1.2 for aromatic H and 1.5 for all other H atoms. Methyl group C34 undergoes considerable thermal motion but splitting the atom did not improve the situation.

### Crystal data

**Table:**

C_12_H_12_N_2_O_2_	*F*(000) = 912
*M**_r_* = 216.24	*D*_x_ = 1.304 Mg m^−^^3^
Monoclinic, *P*2_1_/*n*	Mo *K*α radiation, λ = 0.71073 Å
Hall symbol: -P 2yn	Cell parameters from 4106 reflections
*a* = 13.8735 (7) Å	θ = 2.3–22.4°
*b* = 9.2037 (4) Å	µ = 0.09 mm^−^^1^
*c* = 18.3702 (8) Å	*T* = 296 K
β = 110.100 (2)°	Block, colorless
*V* = 2202.78 (18) Å^3^	0.34 × 0.22 × 0.16 mm
*Z* = 8	

### Data collection

**Table:**

Bruker Kappa APEXII CCD diffractometer	2656 reflections with *I* > 2σ(*I*)
Radiation source: fine-focus sealed tube	*R*_int_ = 0.047
graphite	θ_max_ = 28.6°, θ_min_ = 2.4°
φ and ω scans	*h* = −18→18
24519 measured reflections	*k* = −12→12
5500 independent reflections	*l* = −24→24

### Refinement

**Table:**

Refinement on *F*^2^	Secondary atom site location: difference Fourier map
Least-squares matrix: full	Hydrogen site location: inferred from neighbouring sites
*R*[*F*^2^ > 2σ(*F*^2^)] = 0.051	H-atom parameters constrained
*wR*(*F*^2^) = 0.149	*w* = 1/[σ^2^(*F*_o_^2^) + (0.0571*P*)^2^ + 0.4951*P*] where *P* = (*F*_o_^2^ + 2*F*_c_^2^)/3
*S* = 0.97	(Δ/σ)_max_ = 0.001
5500 reflections	Δρ_max_ = 0.18 e Å^−^^3^
292 parameters	Δρ_min_ = −0.14 e Å^−^^3^
0 restraints	Extinction correction: *SHELXL97* (Sheldrick, 2008), Fc^*^=kFc[1+0.001xFc^2^λ^3^/sin(2θ)]^-1/4^
Primary atom site location: structure-invariant direct methods	Extinction coefficient: 0.0059 (9)

### Special details

**Table:**

Geometry. Bond distances, angles *etc*. have been calculated using the rounded fractional coordinates. All su's are estimated from the variances of the (full) variance-covariance matrix. The cell e.s.d.'s are taken into account in the estimation of distances, angles and torsion angles
Refinement. Refinement of *F*^2^ against ALL reflections. The weighted *R*-factor *wR* and goodness of fit *S* are based on *F*^2^, conventional *R*-factors *R* are based on *F*, with *F* set to zero for negative *F*^2^. The threshold expression of *F*^2^ > σ(*F*^2^) is used only for calculating *R*-factors(gt) *etc*. and is not relevant to the choice of reflections for refinement. *R*-factors based on *F*^2^ are statistically about twice as large as those based on *F*, and *R*- factors based on ALL data will be even larger.

### Fractional atomic coordinates and isotropic or equivalent isotropic displacement parameters (Å^2^)

**Table:**

	*x*	*y*	*z*	*U*_iso_*/*U*_eq_	
O1	0.66613 (12)	0.62373 (18)	−0.03281 (8)	0.0712 (6)	
O2	0.53649 (13)	0.8021 (2)	−0.11913 (9)	0.0837 (7)	
N1	0.62925 (12)	0.57325 (17)	0.08026 (8)	0.0485 (5)	
N2	0.55538 (12)	0.61513 (18)	0.11211 (9)	0.0540 (6)	
C3	0.49372 (15)	0.7059 (2)	0.06310 (11)	0.0526 (6)	
C4	0.52380 (15)	0.7271 (2)	−0.00295 (10)	0.0512 (7)	
C5	0.61166 (15)	0.6395 (2)	0.01114 (10)	0.0514 (7)	
C6	0.70789 (14)	0.4754 (2)	0.12184 (10)	0.0480 (6)	
C7	0.77805 (16)	0.4233 (3)	0.09027 (12)	0.0651 (8)	
C8	0.85362 (18)	0.3279 (3)	0.13225 (13)	0.0744 (9)	
C9	0.85957 (17)	0.2840 (3)	0.20516 (12)	0.0668 (8)	
C10	0.78986 (16)	0.3365 (2)	0.23610 (11)	0.0617 (7)	
C11	0.71414 (15)	0.4321 (2)	0.19540 (10)	0.0547 (7)	
C12	0.48768 (17)	0.8096 (2)	−0.07168 (12)	0.0612 (7)	
C13	0.39531 (18)	0.9041 (3)	−0.09255 (13)	0.0753 (9)	
C14	0.40749 (17)	0.7731 (3)	0.08202 (13)	0.0771 (9)	
O21	0.23044 (14)	0.0334 (2)	0.01074 (9)	0.0987 (8)	
O22	0.18315 (17)	0.1293 (3)	−0.12625 (11)	0.1230 (10)	
N21	0.11930 (13)	0.1209 (2)	0.07053 (9)	0.0632 (7)	
N22	0.03141 (16)	0.2078 (3)	0.04976 (11)	0.0871 (9)	
C23	0.01369 (18)	0.2479 (3)	−0.02204 (13)	0.0760 (9)	
C24	0.08731 (16)	0.1905 (2)	−0.05120 (11)	0.0617 (8)	
C25	0.15261 (17)	0.1086 (2)	0.01064 (12)	0.0622 (8)	
C26	0.15670 (17)	0.0590 (2)	0.14600 (11)	0.0591 (7)	
C27	0.2554 (2)	0.0047 (3)	0.17540 (14)	0.0830 (10)	
C28	0.2890 (2)	−0.0560 (3)	0.24905 (15)	0.0940 (11)	
C29	0.2273 (2)	−0.0600 (3)	0.29305 (14)	0.0882 (10)	
C30	0.1312 (2)	−0.0014 (3)	0.26406 (14)	0.0818 (10)	
C31	0.09504 (19)	0.0573 (3)	0.19051 (12)	0.0706 (8)	
C32	0.1070 (2)	0.2010 (3)	−0.12067 (13)	0.0786 (10)	
C33	0.0467 (2)	0.2899 (3)	−0.18792 (13)	0.0988 (11)	
C34	−0.0776 (2)	0.3424 (4)	−0.06259 (17)	0.1267 (14)	
H1O	0.64280	0.67500	−0.07140	0.1070*	
H7	0.77450	0.45230	0.04090	0.0780*	
H8	0.90090	0.29290	0.11090	0.0890*	
H9	0.91040	0.21950	0.23310	0.0800*	
H10	0.79360	0.30710	0.28550	0.0740*	
H11	0.66750	0.46730	0.21730	0.0660*	
H13A	0.38720	0.95360	−0.14030	0.1130*	
H13B	0.40330	0.97420	−0.05220	0.1130*	
H13C	0.33570	0.84560	−0.09860	0.1130*	
H14A	0.34390	0.75280	0.04120	0.1160*	
H14B	0.41750	0.87630	0.08720	0.1160*	
H14C	0.40560	0.73360	0.12990	0.1160*	
H21O	0.23880	0.04250	−0.03110	0.1480*	
H27	0.29860	0.00880	0.14640	0.1000*	
H28	0.35480	−0.09470	0.26880	0.1130*	
H29	0.25040	−0.10200	0.34210	0.1060*	
H30	0.08950	−0.00120	0.29430	0.0980*	
H31	0.02910	0.09560	0.17120	0.0850*	
H33A	0.06630	0.26630	−0.23180	0.1480*	
H33B	0.05960	0.39110	−0.17560	0.1480*	
H33C	−0.02510	0.27020	−0.20000	0.1480*	
H34A	−0.11410	0.36330	−0.02790	0.1910*	
H34B	−0.12220	0.29250	−0.10740	0.1910*	
H34C	−0.05460	0.43150	−0.07830	0.1910*	

### Atomic displacement parameters (Å^2^)

**Table:**

	*U*^11^	*U*^22^	*U*^33^	*U*^12^	*U*^13^	*U*^23^
O1	0.0762 (10)	0.0911 (12)	0.0547 (8)	0.0196 (9)	0.0331 (8)	0.0127 (8)
O2	0.0920 (12)	0.1028 (14)	0.0569 (9)	0.0153 (10)	0.0262 (9)	0.0176 (8)
N1	0.0489 (9)	0.0560 (10)	0.0417 (8)	0.0082 (8)	0.0170 (7)	−0.0040 (7)
N2	0.0520 (10)	0.0624 (11)	0.0501 (9)	0.0100 (9)	0.0207 (8)	−0.0060 (8)
C3	0.0503 (11)	0.0557 (12)	0.0498 (10)	0.0059 (10)	0.0145 (9)	−0.0072 (10)
C4	0.0511 (12)	0.0513 (12)	0.0461 (10)	0.0036 (10)	0.0101 (9)	−0.0066 (9)
C5	0.0543 (12)	0.0567 (12)	0.0429 (10)	−0.0004 (10)	0.0165 (9)	−0.0060 (9)
C6	0.0478 (11)	0.0500 (11)	0.0460 (10)	0.0046 (9)	0.0157 (9)	−0.0041 (9)
C7	0.0678 (14)	0.0804 (16)	0.0544 (11)	0.0209 (13)	0.0305 (11)	0.0094 (11)
C8	0.0685 (15)	0.0919 (18)	0.0725 (14)	0.0327 (14)	0.0366 (12)	0.0133 (13)
C9	0.0623 (14)	0.0737 (15)	0.0630 (13)	0.0189 (12)	0.0199 (11)	0.0076 (11)
C10	0.0686 (14)	0.0690 (14)	0.0478 (10)	0.0116 (12)	0.0203 (10)	0.0043 (10)
C11	0.0574 (12)	0.0613 (13)	0.0498 (10)	0.0076 (11)	0.0239 (9)	−0.0034 (9)
C12	0.0645 (14)	0.0599 (13)	0.0499 (11)	−0.0007 (11)	0.0078 (11)	−0.0041 (10)
C13	0.0745 (16)	0.0677 (15)	0.0679 (14)	0.0120 (13)	0.0041 (12)	0.0042 (11)
C14	0.0697 (15)	0.0915 (18)	0.0737 (14)	0.0268 (14)	0.0293 (12)	−0.0001 (13)
O21	0.0956 (13)	0.1380 (17)	0.0771 (11)	0.0562 (12)	0.0485 (10)	0.0161 (11)
O22	0.1323 (17)	0.178 (2)	0.0862 (12)	0.0505 (16)	0.0727 (13)	0.0229 (13)
N21	0.0616 (11)	0.0788 (13)	0.0554 (10)	0.0240 (10)	0.0282 (9)	0.0052 (9)
N22	0.0824 (14)	0.1190 (18)	0.0700 (12)	0.0519 (13)	0.0393 (11)	0.0221 (12)
C23	0.0722 (15)	0.0956 (18)	0.0634 (13)	0.0270 (14)	0.0275 (12)	0.0125 (13)
C24	0.0601 (13)	0.0746 (15)	0.0528 (11)	0.0049 (12)	0.0226 (10)	0.0014 (10)
C25	0.0618 (13)	0.0703 (15)	0.0607 (12)	0.0113 (12)	0.0292 (11)	−0.0019 (11)
C26	0.0674 (14)	0.0600 (13)	0.0524 (11)	0.0121 (11)	0.0239 (11)	−0.0007 (10)
C27	0.0782 (17)	0.102 (2)	0.0700 (15)	0.0283 (15)	0.0272 (13)	0.0093 (14)
C28	0.0888 (19)	0.103 (2)	0.0754 (17)	0.0238 (17)	0.0092 (16)	0.0120 (15)
C29	0.112 (2)	0.0838 (19)	0.0616 (14)	−0.0010 (17)	0.0206 (16)	0.0095 (13)
C30	0.107 (2)	0.0783 (17)	0.0679 (15)	−0.0045 (16)	0.0400 (15)	0.0041 (13)
C31	0.0774 (16)	0.0741 (15)	0.0651 (13)	0.0103 (13)	0.0306 (12)	0.0031 (12)
C32	0.0825 (17)	0.0944 (19)	0.0616 (14)	−0.0036 (15)	0.0283 (13)	−0.0008 (13)
C33	0.111 (2)	0.118 (2)	0.0601 (14)	−0.0131 (19)	0.0201 (14)	0.0143 (15)
C34	0.114 (2)	0.177 (3)	0.093 (2)	0.084 (2)	0.0405 (18)	0.042 (2)

### Geometric parameters (Å, °)

**Table:**

O1—C5	1.290 (3)	C11—H11	0.9300
O2—C12	1.276 (3)	C13—H13B	0.9600
O1—H1O	0.8200	C13—H13A	0.9600
O21—C25	1.282 (3)	C13—H13C	0.9600
O22—C32	1.279 (4)	C14—H14C	0.9600
O21—H21O	0.8200	C14—H14B	0.9600
N1—C5	1.353 (2)	C14—H14A	0.9600
N1—C6	1.419 (2)	C23—C24	1.409 (3)
N1—N2	1.398 (2)	C23—C34	1.506 (4)
N2—C3	1.308 (3)	C24—C25	1.404 (3)
N21—C25	1.337 (3)	C24—C32	1.397 (3)
N21—C26	1.422 (2)	C26—C31	1.372 (3)
N21—N22	1.397 (3)	C26—C27	1.381 (4)
N22—C23	1.309 (3)	C27—C28	1.388 (4)
C3—C14	1.490 (3)	C28—C29	1.365 (4)
C3—C4	1.426 (3)	C29—C30	1.365 (4)
C4—C12	1.410 (3)	C30—C31	1.380 (3)
C4—C5	1.409 (3)	C32—C33	1.478 (3)
C6—C11	1.383 (2)	C27—H27	0.9300
C6—C7	1.379 (3)	C28—H28	0.9300
C7—C8	1.382 (4)	C29—H29	0.9300
C8—C9	1.374 (3)	C30—H30	0.9300
C9—C10	1.367 (3)	C31—H31	0.9300
C10—C11	1.377 (3)	C33—H33A	0.9600
C12—C13	1.486 (3)	C33—H33B	0.9600
C7—H7	0.9300	C33—H33C	0.9600
C8—H8	0.9300	C34—H34A	0.9600
C9—H9	0.9300	C34—H34B	0.9600
C10—H10	0.9300	C34—H34C	0.9600
			
C5—O1—H1O	110.00	H14A—C14—H14B	109.00
C25—O21—H21O	110.00	H14A—C14—H14C	110.00
N2—N1—C5	110.30 (16)	C3—C14—H14B	109.00
C5—N1—C6	130.45 (17)	C3—C14—H14C	109.00
N2—N1—C6	119.25 (14)	C3—C14—H14A	109.00
N1—N2—C3	106.75 (15)	N22—C23—C24	111.6 (2)
C25—N21—C26	130.94 (19)	N22—C23—C34	119.8 (2)
N22—N21—C26	119.17 (17)	C24—C23—C34	128.7 (2)
N22—N21—C25	109.87 (17)	C23—C24—C25	104.13 (19)
N21—N22—C23	106.3 (2)	C23—C24—C32	135.7 (2)
C4—C3—C14	129.42 (18)	C25—C24—C32	120.1 (2)
N2—C3—C14	119.48 (18)	O21—C25—C24	126.9 (2)
N2—C3—C4	111.08 (18)	N21—C25—C24	108.1 (2)
C3—C4—C5	104.66 (16)	O21—C25—N21	125.02 (19)
C3—C4—C12	135.9 (2)	N21—C26—C27	120.5 (2)
C5—C4—C12	119.44 (19)	C27—C26—C31	120.0 (2)
N1—C5—C4	107.21 (17)	N21—C26—C31	119.5 (2)
O1—C5—C4	127.25 (17)	C26—C27—C28	118.9 (2)
O1—C5—N1	125.54 (18)	C27—C28—C29	121.3 (3)
N1—C6—C11	119.18 (17)	C28—C29—C30	119.0 (2)
C7—C6—C11	119.64 (19)	C29—C30—C31	121.0 (3)
N1—C6—C7	121.18 (17)	C26—C31—C30	119.8 (2)
C6—C7—C8	119.7 (2)	O22—C32—C33	117.7 (2)
C7—C8—C9	120.7 (2)	C24—C32—C33	124.7 (2)
C8—C9—C10	119.2 (2)	O22—C32—C24	117.6 (2)
C9—C10—C11	121.10 (19)	C26—C27—H27	121.00
C6—C11—C10	119.65 (19)	C28—C27—H27	121.00
O2—C12—C13	117.93 (19)	C27—C28—H28	119.00
O2—C12—C4	118.3 (2)	C29—C28—H28	119.00
C4—C12—C13	123.8 (2)	C28—C29—H29	120.00
C8—C7—H7	120.00	C30—C29—H29	120.00
C6—C7—H7	120.00	C29—C30—H30	120.00
C9—C8—H8	120.00	C31—C30—H30	120.00
C7—C8—H8	120.00	C26—C31—H31	120.00
C10—C9—H9	120.00	C30—C31—H31	120.00
C8—C9—H9	120.00	C32—C33—H33A	110.00
C9—C10—H10	119.00	C32—C33—H33B	109.00
C11—C10—H10	119.00	C32—C33—H33C	109.00
C10—C11—H11	120.00	H33A—C33—H33B	109.00
C6—C11—H11	120.00	H33A—C33—H33C	110.00
C12—C13—H13B	109.00	H33B—C33—H33C	109.00
C12—C13—H13C	109.00	C23—C34—H34A	109.00
H13A—C13—H13C	110.00	C23—C34—H34B	109.00
C12—C13—H13A	109.00	C23—C34—H34C	109.00
H13A—C13—H13B	109.00	H34A—C34—H34B	109.00
H13B—C13—H13C	109.00	H34A—C34—H34C	110.00
H14B—C14—H14C	109.00	H34B—C34—H34C	110.00
			
C5—N1—N2—C3	−0.1 (2)	C3—C4—C5—N1	0.3 (2)
C6—N1—N2—C3	179.20 (16)	C12—C4—C5—O1	−0.2 (3)
N2—N1—C5—O1	179.96 (19)	C12—C4—C5—N1	179.92 (16)
N2—N1—C5—C4	−0.1 (2)	C3—C4—C12—O2	179.4 (2)
C6—N1—C5—O1	0.8 (3)	N1—C6—C7—C8	−179.9 (2)
C6—N1—C5—C4	−179.31 (18)	C11—C6—C7—C8	0.3 (3)
N2—N1—C6—C7	175.42 (19)	N1—C6—C11—C10	179.64 (18)
N2—N1—C6—C11	−4.7 (3)	C7—C6—C11—C10	−0.5 (3)
C5—N1—C6—C7	−5.4 (3)	C6—C7—C8—C9	0.1 (4)
C5—N1—C6—C11	174.41 (19)	C7—C8—C9—C10	−0.3 (4)
N1—N2—C3—C4	0.3 (2)	C8—C9—C10—C11	0.1 (4)
N1—N2—C3—C14	−178.50 (18)	C9—C10—C11—C6	0.4 (3)
N22—N21—C25—O21	−178.4 (2)	N22—C23—C24—C25	0.5 (3)
N22—N21—C25—C24	1.0 (2)	N22—C23—C24—C32	−177.7 (3)
C26—N21—C25—O21	−0.3 (4)	C34—C23—C24—C25	−178.6 (3)
C26—N21—C25—C24	179.2 (2)	C34—C23—C24—C32	3.1 (5)
N22—N21—C26—C27	−163.5 (2)	C23—C24—C25—O21	178.5 (2)
C25—N21—N22—C23	−0.7 (3)	C23—C24—C25—N21	−1.0 (2)
C26—N21—N22—C23	−179.1 (2)	C32—C24—C25—O21	−2.9 (3)
C25—N21—C26—C31	−163.4 (2)	C32—C24—C25—N21	177.6 (2)
N22—N21—C26—C31	14.6 (3)	C23—C24—C32—O22	−179.3 (3)
C25—N21—C26—C27	18.5 (3)	C23—C24—C32—C33	1.3 (5)
N21—N22—C23—C24	0.1 (3)	C25—C24—C32—O22	2.7 (4)
N21—N22—C23—C34	179.3 (2)	C25—C24—C32—C33	−176.8 (2)
C14—C3—C4—C5	178.3 (2)	N21—C26—C27—C28	−179.5 (2)
C14—C3—C4—C12	−1.3 (4)	C31—C26—C27—C28	2.4 (4)
N2—C3—C4—C12	−179.9 (2)	N21—C26—C31—C30	−179.4 (2)
N2—C3—C4—C5	−0.3 (2)	C27—C26—C31—C30	−1.3 (4)
C3—C4—C5—O1	−179.81 (19)	C26—C27—C28—C29	−1.4 (4)
C3—C4—C12—C13	−1.2 (4)	C27—C28—C29—C30	−0.8 (4)
C5—C4—C12—O2	−0.1 (3)	C28—C29—C30—C31	2.0 (4)
C5—C4—C12—C13	179.3 (2)	C29—C30—C31—C26	−0.9 (4)

### Hydrogen-bond geometry (Å, °)

**Table:**

*D*—H···*A*	*D*—H	H···*A*	*D*···*A*	*D*—H···*A*
O1—H1O···O2	0.82	1.85	2.546 (2)	142
O21—H21O···O22	0.82	1.83	2.531 (3)	142
C8—H8···N22^i^	0.93	2.56	3.489 (3)	177
C13—H13C···Cg2^ii^	0.96	2.66	3.533 (3)	150
C33—H33B···Cg2^ii^	0.96	2.98	3.774 (3)	141

Symmetry codes: (i) *x*+1, *y*, *z*; (ii) −*x*+1, −*y*+1, −*z*+1.
